# Responding to a Pandemic Through Social and Behavior Change Communication: Nigeria's Experience

**DOI:** 10.1089/hs.2020.0151

**Published:** 2021-04-08

**Authors:** Chinwe Lucia Ochu, Oluwatosin Wuraola Akande, Vivianne Ihekweazu, Chijioke Kaduru, Oreoluwa Akomolafe, Abiodun Egwuenu, Chimezie Anueyiagu, Jeremiah Agenyi, Ukwori Ejibe, Yinka Falola-Anoemuah, Olayinka Umar-Farouk, Oyeronke Oyebanji, Babafunke Fagbemi, Chukwuemeka Oguanuo, Tijesu Ojumu, Hadiza Saad, Tarik Mohammed, Yahaya Disu, Chikwe Ihekweazu

**Affiliations:** Chinwe Lucia Ochu, MPH, is Acting Director; Abiodun Egwuenu, MPH, is an Epidemiologist; Chimezie Anueyiagu is a Health Education Officer; Chukwuemeka Oguanuo is Corporate Communications, External Comms Lead; and Yahaya Disu, MPH, is Technical Assistant to the Director General on Operations; all in Prevention, Programmes & Knowledge Management, Nigeria Centre for Disease Control, Abuja, Nigeria. Oreoluwa Akomolafe, MSc, is Technical Assistant to the Director General; Jeremiah Agenyi is a Communication Consultant, Corporate Communications; Oyeronke Oyebanji is a Strategy Coordinator; Tijesu Ojumu is Corporate Communications, Multimedia Lead; Tarik Mohammed is a Scientific Officer; and Chikwe Ihekweazu is Director General; all in the Office of the Director General, Nigeria Centre for Disease Control, Abuja, Nigeria. Hadiza Saad, MPH, is a Scientific Office, Surveillance and Epidemiology, also with Nigeria Centre for Disease Control, Abuja, Nigeria. Oluwatosin Wuraola Akande, MWACP, is a Public Health Physician, Epidemiology and Community Health; University of Ilorin Teaching Hospital, Kwara State, Nigeria. Vivianne Ihekweazu, MSc, is Managing Director, Management, Nigeria Health Watch; Chijioke Kaduru, MPH, is Technical Director, Health Division, Corona Management Systems; Ukwori Ejibe, MPA, is Governance Advisor, Strategic Communications and Partnerships, Tony Blair Institute for Global Change; Yinka Falola-Anoemuah, PhD, is Deputy Director and Lead, Gender Human Rights and Care Support Services, Community Prevention and Care Services, National Agency for the Control of AIDS; Olayinka Umar-Farouk, MBA, is Senior Technical Advisor, Management, Breakthrough ACTION Nigeria; and Babafunke Fagbemi, MBA, is Executive Director, Management, Centre for Communication and Social Impact; all in Abuja, Nigeria.

**Keywords:** COVID-19, Social and Behavior Change Communication, Risk communication

Emerging and reemerging infectious diseases with potential to cause disease outbreaks form majority of public health challenges experienced globally.^1^ The 21st century has experienced several infectious disease outbreaks and pandemics, such as severe acute respiratory syndrome (2003), H1N1 influenza (2009), Middle East respiratory syndrome (2013), Ebola virus disease (2014-2016 West Africa and 2018-2020 Democratic Republic of the Congo), and now coronavirus disease 2019 (COVID-19).^2,3^ The COVID-19 pandemic has reiterated the need for multidimensional approaches in fighting pandemics, including the integration of social science into emergency risk communication.

Nigeria's tropical climate, population density, and poor socioeconomic indicators place the country at risk of annual disease outbreaks.^4,5^ In the last 4 years, Nigeria has experienced outbreaks of Lassa fever, yellow fever, monkeypox, cholera, and other infectious diseases.^6^ These outbreaks and the more recent COVID-19 pandemic have highlighted the critical need for risk communication as an integral component of outbreak response.

Social and behavior change communication (SBCC) is the methodical use of theory-based, evidence-based communication strategies to address change at the individual, environmental, and structural levels.^7^ It is an influential strategy used to recognize and understand the social determinants of people's behaviors and how these behaviors can be collectively shaped to achieve positive action.^8^

The goal of SBCC interventions in pandemic preparedness and response is to reduce morbidity and mortality by preventing transmission among those who are well and those caring for the sick and by preventing further transmission of the disease among those who are sick.^9^ SBCC strategies include a variety of risk communication interventions that can be carried out through interpersonal communication, community engagement, and mass media.

Although the 2016 Infectious Disease Vulnerability Index identifies that 22 out of the 25 countries most vulnerable to infectious disease outbreaks are in Africa, the COVID-19 pandemic on the African continent remains considerably less severe than predicted.^10^ Lessons in communicating risk are usually retroactive,^11^ yet the African continent has developed significant experience over the last decade in effective communications during outbreaks. There are many allusions to the role of this experience in the management of the COVID-19 outbreak response.^12^

The experience in communications from the Ebola outbreaks in West and Central Africa and other recent outbreaks particularly provide key lessons on the need for a strategic and responsive approach for effective communications during the pandemic.^11^ Case reports from other countries highlight the key role of communication in the COVID-19 response,^13,14^ with countries adopting a wide range of strategies to strengthen risk communications systems, improve public communications, execute dynamic listening, and manage rumors, as well as to coordinate partners and stakeholders, placing communities at the heart of the response.

This paper aims to share the Nigerian experience in relation to SBCC interventions by the Nigeria Centre for Disease Control (NCDC) and partners during the COVID-19 pandemic and to highlight lessons learned.

## SBCC Strategies in Nigeria

There has been a wide range of perceptions and emotions exhibited by people since the beginning of the COVID-19 pandemic. Fear, anxiety, distrust, and denial are all community perceptions and emotions that have characterized this pandemic.^16,17^ Nonpharmaceutical interventions have been prioritized globally as control measures for COVID-19, in the absence of an effective vaccine.^18^ The introduction of these interventions has required rapid behavior change through community engagement at all levels of the society.

The NCDC leads the public health response to the COVID-19 outbreak, through the national Emergency Operations Centre (EOC).^19^ A risk communication and community engagement strategy for responding to the COVID-19 outbreak was developed by NCDC through robust multistakeholder collaboration. This strategy formed the operational roadmap of the Risk Communication Pillar of the national EOC. The foundation of the strategy was built on the 5 pillars of risk communications recommended by the World Health Organization^20^ and it employed an Extended Parallel Process Model theoretical framework.^21^ The strategy outlined SBCC interventions and approaches as a means of encouraging healthy individuals to adopt and maintain healthy behaviors grounded on evidence-based scientific knowledge.^22^

An array of health promotion theories are considered when implementing SBCC and risk communication approaches, with a shift from traditional narrowly conceived behavioral change models—which is characterized by victim-blaming and is most times ineffective—to self-empowerment and socioecological models that empower individuals to take control of their health, taking into account the interrelationship of individuals and their physical, socioeconomic, and political environment.^23,24^

At the beginning of the pandemic and following confirmation of the first cases of COVID-19 in Nigeria, a top-down approach was used in the conceptualization and dissemination of risk communication messages by the NCDC. This was necessitated by the emergency nature of the outbreak. This approach required engaging with the Nigerian population, building trust, and disseminating messaging using a multimedia approach. Uniform messages were developed at the national level and disseminated to subnational levels for adaptation. This approach was not effective and did not lead to the desired behavioral change outcomes. The novelty of COVID-19 and its unprecedented spread, the overwhelming opportunities for misinformation, as well as a myriad of contributing sociocultural, political, and economic realities required an approach that factored in the complexity of social norms that influence behavioral change. A review of the strategy led to the adoption of a bottom-up approach in May 2020, when the country entered into the community transmission phase of the pandemic.^25^ Using this approach, communities were empowered to identify the risk communication challenges in their localities, proffer solutions, and develop their own risk communication messages.^26^ This implied that risk communication messages now differed in structure across various communities in the country, as these messages became more contextualized. The Risk Communication Pillar of the national EOC of the NCDC and the Risk Communication Unit of the Presidential Task Force on COVID-19, with the support of the government of the United Kingdom and NOI polls, provide weekly updates on risk communication and community engagement in some states in Nigeria.^27^ In Lagos state, data from the polls conducted in June 2020 revealed that less than two-thirds (59%) of 554 participants thought the COVID-19 was real (NCDC, unpublished data, June 2020). By August 2020, this percentage had increased to 81%, about 12 weeks into the implementation of the bottom-up approach (NCDC, unpublished data, August 2020). More communities across the country are being supported to develop their risk communication messages, identify strategies for dissemination, and implement these strategies.

## Lessons Learned from SBCC

The lessons learned can be categorized based on these 4Cs of communication: content, communicator, context, and channel.

### Content

Given the novel nature of COVID-19 and limited point of reference, the response to this pandemic has been characterized by evolving scientific advice as new knowledge and evidence emerge. These factors have contributed to an unusual level of instability in communication messages, creating opportunities for mistrust, misinformation, and confusion among the public, who may not clearly understand the nuances and evolving nature of science. For instance, the use of face masks during the initial phase of the COVID-19 pandemic among healthy individuals was neither recommended nor adopted globally.^28^ However, as the pandemic progressed, scientific evidence regarding the effectiveness of masks in combination with other nonpharmaceutical public health measures among healthy individuals started to emerge, and health authorities began to include the wearing of masks in public health advisories.^29,30^

A further lesson from the pandemic was the challenge posed by the acceptability of required behavioral change among the Nigerian population. In a country with a rich social capital, characterized by regular social gatherings and celebrations, the recommendation of physical distancing as a means of curbing the pandemic was met with some resistance. The wearing of face masks was seen as unnatural and uncomfortable with resulting widespread noncompliance.

Convincing people to adopt these new measures demands an empathetic approach and calls for altruism. The choice of adopting a new and difficult behavior is dependent on the level of risk perception of the person involved and confidence in the prescribed strategies for mitigating the risk. An effective communication message should have enough content to increase risk perception while palliating the undesired effect of positive behavior. The risk communicator must empathize with the target individuals or communities. This is in addition to providing feasible and acceptable alternatives to the practices being discouraged.

Communicating socio-behavioral change without providing an alternative could be disempowering. Effective risk communication must, therefore, be underpinned by the concept of “the other alternative.” We propose that for every desired behavioral change, there should be a reasonable and attainable alternative to the current behavior. Social and behavioral change interventions should provide practicable alternatives and communicate these pragmatically with adequate community participation to increase adoption. Adapting positive behavior in a vacuum without an alternative choice is impracticable. This also brings to fore the importance of a socioecological model for risk communication.^31^ Individual behaviors are usually products of complex wider sociocultural, societal, economic, and political factors that must be considered in seeking to address risky behaviors.

Therefore, a shift from a victim-blaming approach toward individual and community empowerment is necessary for effective SBCC. In Nigeria, the shift in community engagement approach led to greater community participation and ownership; members of the target audience were involved in drafting messages and infographics, animations, and radio jingles tailored to their community at the latter phase of the response.

### Communicator

The effectiveness of risk communication is not only defined by the content of the message but also by the communicator of the message. The key questions to be answered include: “Who is this change advocate?” “What is the track record of this change advocate?” “How well does the community trust this communicator?”

The track record and perception of the communicator is critical in the acceptance of the message being conveyed to the community. There is significant mistrust in the Nigerian government by its citizens.^32^ The NCDC is a public health institute and also the government organization responsible for conveying public health messages. Given the importance of its role, NCDC prioritized building up the public's trust through transparent communication. This included daily publication of situation reports, updates on response challenges, and related issues. Due to existing mistrust, this transparency was largely misinterpreted by the public as part of government conspiracy.

From NCDC's experience, the top-down approach in delivering SBCC messages has not been as effective. People have generally responded better to risk communication strategies codeveloped with the target community and conveyed by community gatekeepers and influencers that they can relate with. This has enabled other members of the society—outside of government—to be seen as the communicator.

### Context

SBCC must be tailored to the context of the target community. The same message may result in dissimilar interpretations across different sects of the population. Nigeria is a multicultural country with more than 250 distinct ethnic groups and over 500 languages.^33^ Such sociocultural diversity means that effective communication must be adapted to the relevant context. An acceptable risk communication approach must be culturally sensitive. This further highlights the importance of engaging the target communities, who best understand their local context, in identifying and prioritizing their health problems and working together to develop solutions. A contextual, culturally sensitive community engagement approach is being implemented currently by NCDC and its communication partners in Nigeria.

A good example of sociocultural diversity that can affect risk communication is the governance structure in the various geopolitical zones of the country. For instance, in Northern Nigeria, and to some extent South West Nigeria, the monarchical system of governance is highly revered. Traditional rulers (Emirs and Obas) are considered important custodians of knowledge and trust with great influence on the majority of the population under their jurisdiction. However, in South East Nigeria, among the Igbos, the scenario is quite different. Monarchs are less influential and may not be effective means of SBCC compared to other community influencers, such as age-group leaders, women associations, traders' union, and religious leaders.^34^ A contextual approach in identifying and engaging community influencers is, therefore, required for effective SBCC.

### Channel

Closely related to the context of communication is the channel of communication. The NCDC makes use of both contemporary (ie, social media, blogs, websites) and traditional channels (eg, print media, radio broadcast, television) in communicating evidence-based SBCC messages nationwide. The internet through social media is an effective means of communication for the upwardly mobile, younger, educated, and urban resident audience, while the use of town announcers, radio, and interpersonal communication could be more suitable for the older population and those in rural areas. In Nigeria, the NCDC utilized campaigns on Twitter, Facebook, and Instagram to boost the dissemination of messages on social media. Furthermore, the language of communication was identified as a key indicator for dissemination. The appropriate translation of messages into local dialects is important in ensuring that the target population understand the messages being conveyed. Many risk communication messages for COVID-19 in Nigeria were translated into at least 12 local languages. Factors such as age, gender, socioeconomic class, educational level, religion, and place of residence (rural versus urban) influenced the type of channel used to effectively communicate SBCC messages. These factors, as well as the reach of the channel of communication, should be considered in the selection of appropriate media of communication.

## Recommendations

Lessons from previous outbreaks should be used to prepare for future outbreaks, especially in the context of emergencies that require rapid response. Community members and influencers who best understand their health needs and priorities should be involved in the planning and implementation of SBCC programs. The 4Cs of communication—content, communicator, context, and channel—should be considered when risk communication messages are being developed and disseminated. For risk communication to be effective, it should be supported by the concept of flexibility, adaptability, and “the other alternative.” Furthermore, socioecological models that incorporate sociocultural, economic, and political insights, should guide SBCC strategy development and implementation.

## Conclusion

Nigeria's experience has highlighted the importance of effective community engagement in achieving positive behavioral change through SBCC. The integration of social science through SBCC into the pandemic preparedness and response contributes to sustainable development by building resilience among individuals, communities, and health systems. Risk communication helps to achieve public health outcomes by promoting behavioral change and increasing support for scientific recommendations. The initial response to the COVID-19 pandemic in Nigeria was characterized by emergency risk communication activities that were top-down. As the transmission dynamics evolved, various risk communication and community engagement approaches that are community-driven and based on evolving evidence have been adopted. For SBCC to be effective, it should consider the wider social, societal, and economic determinants of risky behavior. Target communities must be empowered by supporting their participation in ideation, coproduction, and implementation of SBCC interventions so that they have greater ownership of the response. Sustainability in the public health response is critical. This requires sustained funding for risk communication, as well as building the capacity of local communities to maintain public health measures, while also taking responsibility for the response.
